# Experimental Investigation of the Combustion Characteristics and Thermal Hazards of Methylsilyl-Modified Silica Aerogels

**DOI:** 10.3390/gels10110702

**Published:** 2024-10-30

**Authors:** Xiaoxu Wu, Kai Shen, Min Hu, Fang Zhou, Zikang Chen, Qiong Liu, Zijun Li, Zhi Li

**Affiliations:** School of Resources and Safety Engineering, Central South University, Changsha 410083, China235512138@csu.edu.cn (F.Z.); 235512117@csu.edu.cn (Z.C.); liuqiong@csu.edu.cn (Q.L.)

**Keywords:** silica aerogels, methylsilyl, combustion characteristics, thermal hazard

## Abstract

The thermal safety of hydrophobic silica aerogels (SAs) is essential to thermal insulation applications. Herein, trimethylchlorosilane (TMCS), dimethyldichlorosilane (DMDCS), and methyltrichlorosilane (MTCS) were employed as surface modifiers to prepare three different methylsilyl-modified SAs (i.e., TSA, DSA, and MSA) and their combustion characteristics and thermal hazards were experimentally studied in detail. The cone calorimeter test found that the three SAs have similar combustion processes and the variations in ignition time and fire spread rate with the heat flux obey simple logarithmic and linear relationships, respectively. It further found that TSA has the most methylsilyl groups on silica skeletons and thus has the largest heat release, followed by DSA and MSA in turn, implying that TSA has the greatest fire hazard among the three SAs. These results further demonstrate that the type and quantity of methylsilyl groups on the skeletons of SAs significantly affect the thermal hazard of methylsilyl-modified SAs. In addition, the combustion mechanism of the methylsilyl-modified SAs is discussed. In total, this work experimentally studies the combustion characteristics of methylsilyl-modified SAs and compares their thermal hazards, clarifying the potential fire risk of methylsilyl-modified SAs in practical thermal insulation applications.

## 1. Introduction

Silica aerogels (SAs) have gained considerable attention in recent years due to their unique physicochemical properties, such as low density (0.003–0.500 g/cm^3^), high apparent surface area (500–1200 m^2^/g), and extremely low thermal conductivity (0.014–0.021 W/m/K) [1,2,3,4,5,6]. These characteristics make SAs highly promising in various fields, including gas and liquid adsorption and separation [7,8,9], thermal insulation [10,11,12], catalyst support [13,14], drug delivery systems [15,16], and aerospace applications [17,18]. Among these, thermal insulation is one of the fastest-growing application areas, spanning building energy conservation [19,20], insulation for industrial pipes and tanks [21,22], and thermal runaway prevention in new energy vehicles [23,24]. To ensure long-term thermal insulation performance, hydrophobic SAs are typically used as their hydrophobic groups prevent water vapor intrusion [25].

The synthesis of SAs frequently employs the sol–gel process, a widely used method for producing various gel-like materials, including silica aerogels. The sol–gel process can be used to fabricate materials such as phosphorus- and zirconium-containing gels, tin, titanium, boron gels, and more. These materials have garnered attention due to their potential application across different industries, from thermal insulation to biocompatible medical materials, particularly when zirconium or titanium is involved. Phosphorus-based materials such as polyphosphates and derivatives of phosphoric, phosphonic, and phosphinic acids are of particular interest for their potential use as precursors in sol–gel reactions [26], as is demonstrated in recent studies [27,28]. The sol–gel method not only enables the synthesis of pure silica aerogels but also facilitates the incorporation of functional materials, offering the potential for medical, industrial, and environmental applications.

Methylsilyl groups, commonly introduced via surface derivatization or co-precursor methods, are frequently used to enhance the hydrophobicity of SAs [29,30,31]. Organosilanes such as trimethylchlorosilane (TMCS) [32,33,34] and methyltri(m)ethoxysilane (MTES, MTMS) [35,36,37] can successfully graft methylsilyl groups onto the silica skeletons, conferring excellent hydrophobic properties. However, these organic components also introduce thermal safety concerns, as methylsilyl groups are susceptible to decomposition at high temperatures, releasing heat and flammable gases which pose a fire risk [38]. This potential hazard must be carefully considered, especially in thermal insulation applications where exposure to high heat or flame is common.

Several studies have focused on fire risks associated with hydrophobic SAs. For instance, Wakili et al. [39] explored the fire resistance of aerogels containing ceramic fibers, while He et al. [40] and Zhang et al. [41,42] investigated the pyrolysis behavior of hydrophobic SAs, providing insight into their thermal decomposition kinetics and mechanisms. Additionally, Li et al. [43] studied the combustion behaviors of SAs prepared by different drying methods and confirmed that heat treatment could improve the thermal safety of these materials, making them suitable for high-temperature applications.

Methylsilyl groups are typically classified as trimethylsilyl (TMS), dimethylsilyl (DMS), or monomethylsilyl (MMS) based on the number of methyl groups. Although the introduction of methylsilyl groups significantly enhances hydrophobicity in SAs, their influence on combustion characteristics and thermal hazards have not been systematically studied across different types of methylsilyl groups. This gap in knowledge limits our understanding of the thermal safety characteristics of hydrophobic SAs.

Therefore, this work aims to address this shortcoming by investigating the combustion characteristics and thermal hazards of three types of methylsilyl-modified SAs: trimethylchlorosilane (TMCS), dimethyldichlorosilane (DMDCS), and methyltrichlorosilane (MTCS). We systematically analyze the combustion processes and mechanisms of these materials and compare their thermal hazards to provide a more comprehensive understanding of the fire risk in practical thermal insulation applications.

## 2. Results and Discussion

### 2.1. Basic Physicochemical Properties of SAs

#### 2.1.1. Density and Thermal Conductivity

**Figure 1:**
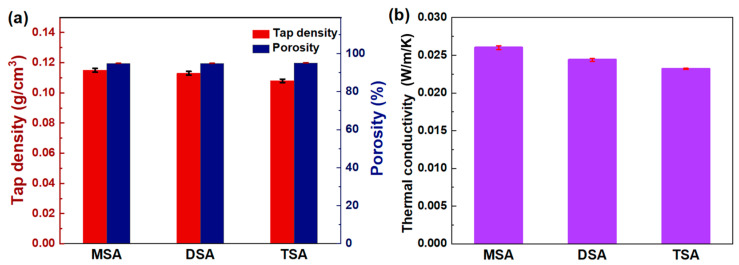
Tap density, porosity, and thermal conductivity in methylsilyl-modified SAs. (**a**) Tap density and porosity, and (**b**) thermal conductivity.

Figure 1a presents the tap density and porosity of various methylsilyl-modified SAs. All three SAs have a low density of ~0.11 g/cm^3^, and a high porosity of ~95%. Note that the SAs modified with TMCS, DMDCS, and MTCS were renamed TSA, DSA, and MSA, respectively. There is a slight decrease in the density of the SAs in the following order: MSA, DSA, and TSA. Their porosity demonstrates an opposite trend. We speculate this to be related to the composition and structure of the grafted methylsilyl groups. In Figure 1b, thermal conductivity is positively related to density, i.e., there is a decrease in the following order: MSA, DSA, and TSA. In general, all three SAs have thermal conductivities lower than static air, which demonstrate excellent thermal insulation performance.

#### 2.1.2. Microstructures and Pore Structure

**Figure 2:**
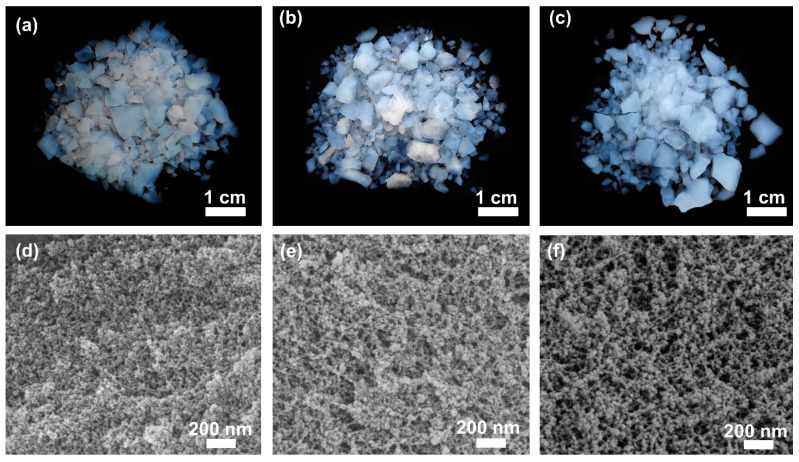
Optical microscopy images (**a**–**c**) and SEM images (**d**–**f**) showing the sample images and microstructure of the methylsilyl-modified SAs: (**a**,**d**) MSA, (**b**,**e**) DSA, and (**c**,**f**) TSA.

Figure 2a–c present the typical physical appearance and microstructures of the methylsilyl-modified SAs. All of the prepared SAs are primarily composed of light blue fragments, while some white fragments are found in MSA and DSA; these white fragments can be removed through multiple solvent cleaning after surface modification. Figure 2d–f show the representative nanoporous skeletal structures of the methylsilyl-modified SAs. We found that MSA had the densest silica skeleton, followed by DSA; TSA had the loosest porous skeletal structure. This difference in silica network structure is caused by the various condensation reactions during the formation of gel skeletons. Furthermore, the variation in microstructure of the three SAs precisely explains the change in density and thermal conductivity.

**Figure 3:**
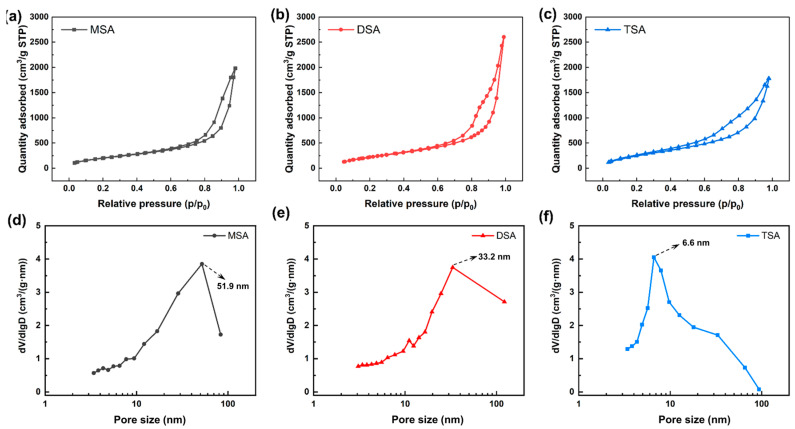
Nitrogen sorption and pore size distribution of methylsilyl-modified SAs. (**a**,**d**) MSA, (**b**,**e**) DSA, and (**c**,**f**) TSA.

Figure 3a–c present the nitrogen sorption of methylsilyl-modified SAs. Three curves have typical type IV characteristics, indicating that the methylsilyl-modified SAs are all mesoporous materials [44]. The hysteresis loop of H3 indicates the presence of a slit pore structure in these SAs. Figure 3d–f are the pore size distributions of the three methylsilyl-modified SAs. The most probable pore sizes of MSA, DSA, and TSA descend from 51.9 nm to 6.6 nm. In Figure 3 and Table 1, as the number of introduced methyl groups increases, the BET surface area also increases, while the average pore size decreases. The pore volume of MSA, DSA, and TSA is 34.2 nm, 38.8 nm, and 41.9 nm, respectively. The difference is due to the number of methyl groups introduced.

#### 2.1.3. FTIR Analysis and Hydrophobicity

**Figure 4:**
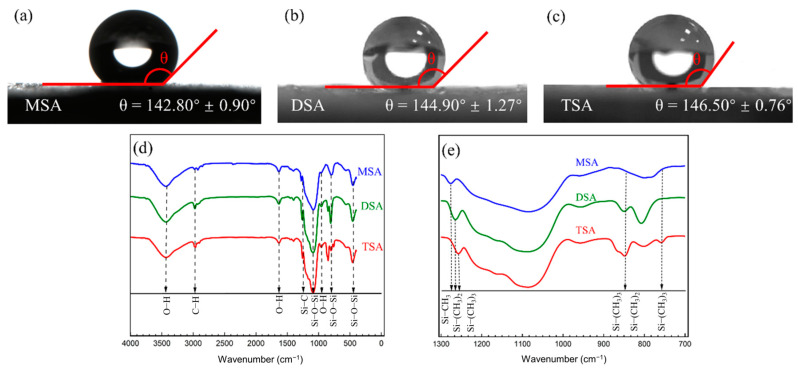
The contact angles of (**a**) MSA, (**b**) DSA, (**c**) TSA, and (**d**,**e**) FTIR spectra of methylsilyl-modified SAs.

Figure 4a–c present the contact angles of three methylsilyl-modified SAs. The measured contact angles of MSA, DSA, and TSA are 142.8°, 144.9°, and 146.5°, respectively, which all indicate the methylsilyl-modified SAs have good hydrophobicity. This property may largely depend on the methyl groups introduced.

The FTIR spectra of three methylsilyl-modified SAs are depicted in Figure 4d,e. The absorption peaks at 3340, 1630, and 950 cm^−1^ are traditionally associated with O-H stretching, bending, and deformation vibrations, respectively. However, after the silylation process, the presence of O-H groups was significantly reduced, as the silyl groups introduced during the modification largely replaced the surface hydroxyl groups. This is consistent with the thermogravimetric analysis, which showed minimal weight loss in the initial stage of the TG curves, indicating that only a small amount of residual O-H groups remained. The presence of these peaks in the FTIR spectra can be attributed to trace amounts of moisture or residual hydroxyl groups [45]. The peaks observed at 460 cm^−1^, 800 cm^−1^, and 1080 cm^−1^ are due to the Si-O-Si bending vibration, symmetric vibration, and asymmetric vibration modes. The sign of successful surface modification in SAs is the existence of C-H bonds and Si-C bonds. The C-H bonds usually appear around 2954 cm^−1^ and 1398 cm^−1^ and the peaks at 1265 cm^−1^ and 845 cm^−1^ correspond to Si-C bonds. We found that MSA had a slightly lower absorption intensity of Si-C bonds than DSA and TSA, which was caused by the quantity of Si-CH_3_ groups.

In order to further analyze the influence of the quantity of Si-CH_3_ groups, the enlarged FTIR spectrum from 700 cm^−1^ to 1300 cm^−1^ is shown in Figure 4e. We found that the absorption peaks of the Si-(CH_3_)_3_ group in TSA are 1255 cm^−1^, 845 cm^−1^, and 755 cm^−1^, respectively. In DSA, the peaks of the Si-(CH_3_)_2_ group are located at 1265 cm^−1^ and 849 cm^−1^. In MSA, the peak of the Si-CH_3_ group appears at 1275 cm^−1^. Combined with the hydrophobic angle of the SAs, it was found that the number of Si-CH_3_ groups was positively correlated with hydrophobicity. As the number of Si-CH_3_ groups increased, the methylsilyl-modified SAs became more hydrophobic.

### 2.2. Combustion Characteristics of SAs

#### 2.2.1. Gross Calorific Value

**Figure 5:**
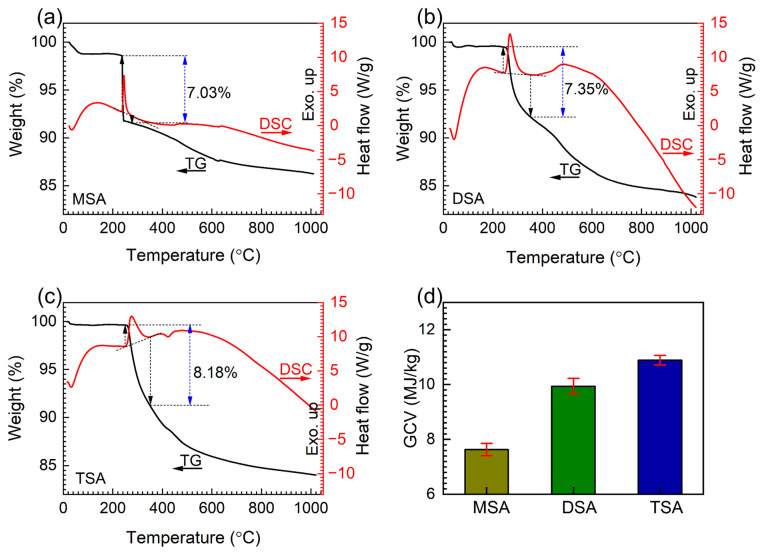
The TG–DSC curves of the methylsilyl-modified SAs (**a**) MSA, (**b**) DSA, and (**c**) TSA, and (**d**) their gross calorific values.

The weight losses of methylsilyl-modified SAs are presented in Figure 5a–c. In each TG curve, the primary weight loss is associated with a clear exothermic peak in the corresponding DSC curve, indicating the thermal decomposition of the methylsilyl groups [46]. The weight losses observed at each stage of the thermal process are summarized as follows: MSA showed a weight loss of 7.03%, DSA showed 7.35%, and TSA showed 8.18%. These weight losses represent the cumulative thermal degradation of the methylsilyl groups across the entire temperature range studied [38]. The increase in weight loss from MSA to TSA reflects the higher quantity of methylsilyl substitution in the samples. Additionally, the gross calorific values (GCVs) presented in Figure 5d also increase in the same order, further confirming that TSA has a significantly higher content of methylsilyl groups compared to MSA. These findings clarify that the weight losses observed correspond to the thermal decomposition of the methylsilyl groups, and their variation across the different samples is consistent with the amount of methyl substitution.

#### 2.2.2. Combustion Process

**Figure 6:**
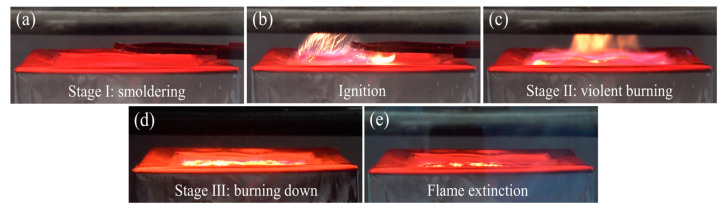
The combustion processes of the methylsilyl-modified SAs at a heat flux of 35 kW/m^2^: (**a**) the smoldering, stage I, (**b**) the ignition moment, (**c**) the violent burning, stage II, (**d**) the burning down, stage III, and (**e**) the flames’ extinction.

We found that the combustion processes of MSA, DSA, and TSA are very similar and can be roughly divided into three stages, namely: a smoldering stage, a violent burning stage, and a burning-down stage, as shown in Figure 6. At the smoldering stage (Figure 6a), the SA powders heat up rapidly and swell slightly when exposed to the radiation cone. Meanwhile, the flammable volatiles produced by the pyrolysis of SAs rise from the gaps in the SA powders. It is reported that these combustible volatiles are mainly generated from thermal decomposition of the methylsilyl groups on the SA skeletons. Upon those combustible volatiles reaching a certain concentration and encountering an electric spark, ignition then takes place (Figure 6b), producing small saffron flames. As the flames spread over the whole surface of the SA powders, the reaction develops into the violent combustion stage (Figure 6c) where the flames grow stronger and the flame color turns to light blue, indicating an increase in the flame temperature. In addition, as the pyrolysis that produced flammable volatiles is gradually consumed, the flames attenuate and enter the burning-down stage (Figure 6). When the flammable volatiles can no longer maintain a continuous combustion the flames eventually extinguish (Figure 6e).

#### 2.2.3. Ignition Time and Critical Heat Flux

**Figure 7:**
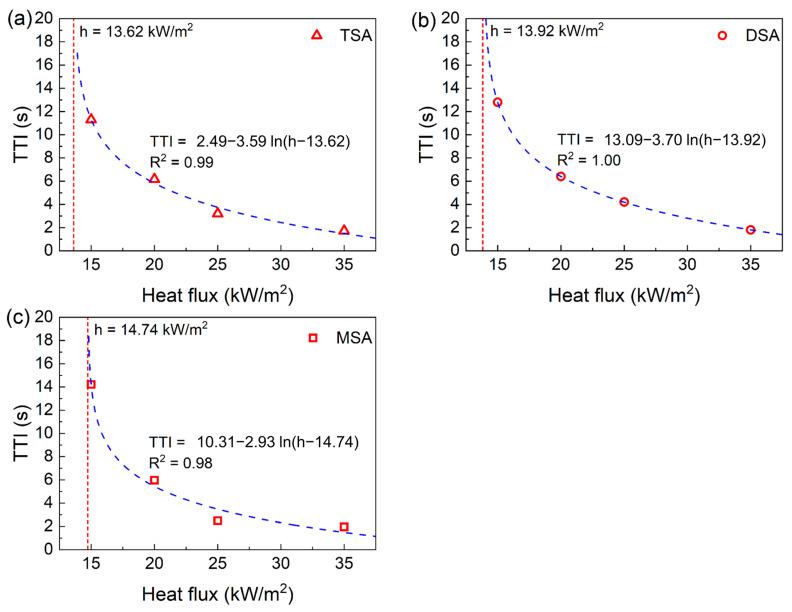
The ignition times of the methylsilyl-modified SAs and their corresponding heat flux: (**a**) TSA, (**b**) DSA, and (**c**) MSA. The fitted equations are presented above, in which TTI is the time to ignition and h is the heat flux.

The ignition time refers to the time from the moment the radiation shield in the cone calorimeter is removed to the time a stable flame appears on the SA powder’s surface. A longer ignition time means the material is less likely to catch fire under external thermal radiation, indicating better fire resistance [47]. The variation in ignition time of the methylsilyl-modified SAs and their corresponding heat flux is presented in Figure 7. We found that the ignition times of TSA, DSA, and MSA all decrease with an increase in radiant heat flux, which are well-fitted to simple logarithmic equations.

Obviously, a larger heat flux will heat up the SA powders faster. Meanwhile, the SAs pyrolyze faster and release flammable gas more quickly. Once they reach a certain concentration, ignition occurs. This causes the ignition time to decrease as the heat flux increases. When the heat flux reaches 35 kW/m^2^, the ignition times of these three methylsilyl-modified SAs are all less than 2 s, indicating a significant fire risk.

The critical heat flux is the radiant heat flow intensity when the ignition time approaches infinity. According to the fitted equations, the critical heat fluxes of TSA, DSA, and MSA are 13.62 kW/m^2^, 13.92 kW/m^2^, and 14.74 kW/m^2^, respectively. When encountering external thermal radiation lower than the corresponding critical heat flux, all three methylsilyl-modified SAs cannot be ignited. Furthermore, from a critical heat flux perspective, it follows that MSA has a lower fire risk than TSA and DSA.

#### 2.2.4. Fire Spread Rate

In the described combustion process of SAs, ignition initially occurs at a localized point and subsequently spreads outward, eventually covering the entire surface. This behavior is analogous to the propagation of a pool fire [48]. To quantitatively characterize the fire spread properties on the surface of the aerogel, we employed the concept of fire propagation in this study and calculated the fire spread rate based on video images, though fire spread rate is typically only determined in large-scale fire experiments.

**Figure 8:**
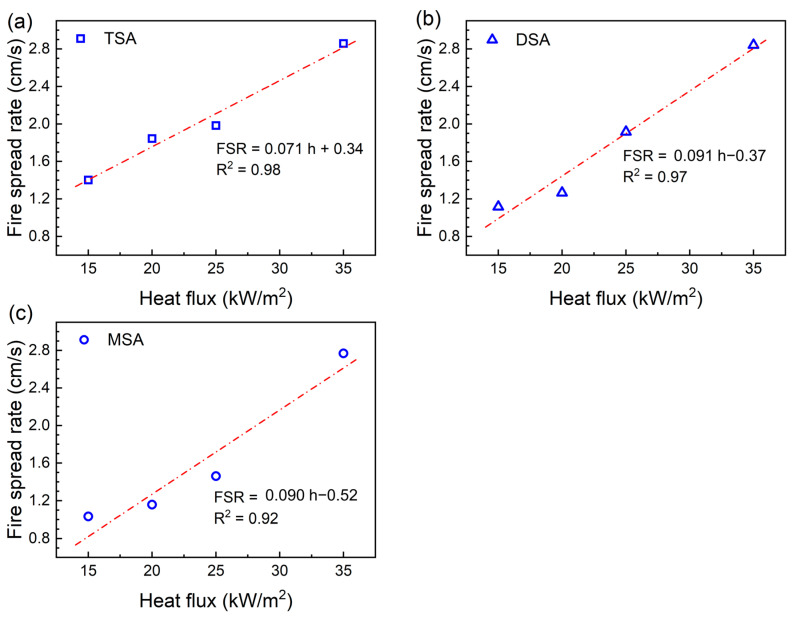
The fire spread rates of the methylsilyl-modified SAs and their corresponding heat flux: (**a**) TSA, (**b**) DSA, and (**c**) MSA. The fitted equations are presented above, in which FSR is the fire spread rate and h is the heat flux.

As depicted in Figure 8, the fire spread rates of the methylsilyl-modified SAs exhibited a consistent upward trend with the increase in heat flux. The linear relationship between these two parameters was further corroborated by additional analyses. At a heat flux below 35 kW/m^2^, TSA exhibited a notably higher fire spread rate compared to DSA and MSA. However, when the heat flux reached 35 kW/m^2^, the fire spread rates of all three materials converged to approximately 2.8 cm/s. This narrowing of the difference in fire spread rate is likely due to the release rates of combustible gases during pyrolysis.

### 2.3. Fire Hazard of SAs

#### 2.3.1. Heat Release

**Figure 9:**
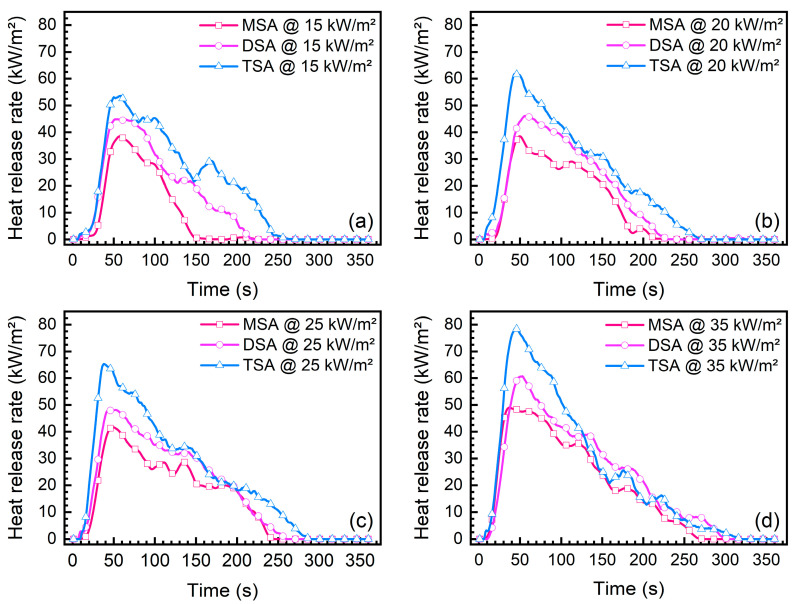
Heat release rates of methylsilyl-modified SAs under various heat fluxes: (**a**) 15 kW/m^2^, (**b**) 20 kW/m^2^, (**c**) 25 kW/m^2^, and (**d**) 35 kW/m^2^.

Figure 9 presents the heat release rates of the methylsilyl-modified SAs under various heat fluxes. The variation in heat release rate corresponds to the combustion process, and the three methylsilyl-modified SAs have similar heat release behavior. Namely, after ignition, the heat release rate dramatically rises up to a peak and then gradually decreases to a negligible value until the flame extinguishes. As the heat flux increases, the heat release rate of each methylsilyl-modified SA also increases, especially at its peak. Furthermore, at the same heat flux, we found that the heat release rate of TSA was usually larger than that of DSA and MSA. The heat release rate represents the heat released by the combustion of a material per unit time under specified test conditions. A greater heat release rate indicates that more heat is released during the combustion process. In this regard, TSA presents the greatest fire hazard of the three SAs and MSA and DSA have a relatively low fire risk.

**Figure 10:**
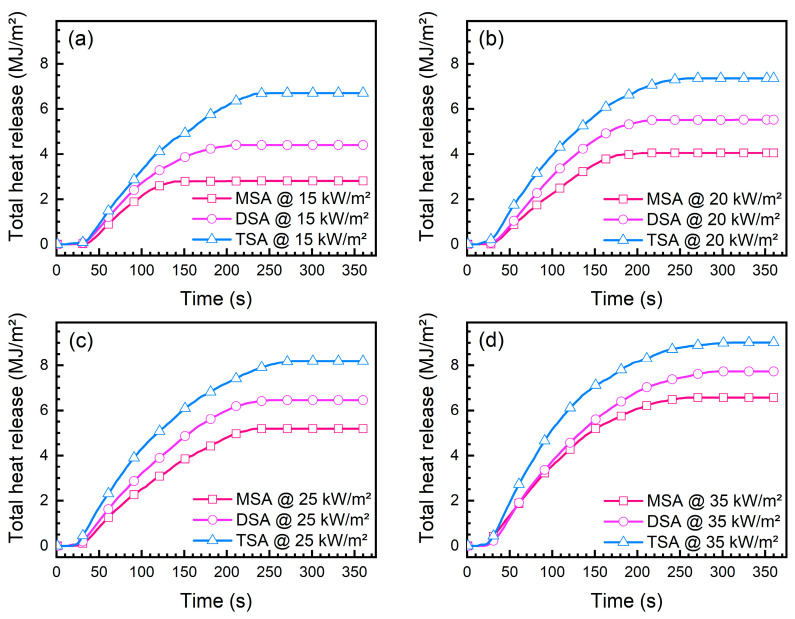
Total heat release of methylsilyl-modified SAs under various heat fluxes: (**a**) 15 kW/m^2^, (**b**) 20 kW/m^2^, (**c**) 25 kW/m^2^, and (**d**) 35 kW/m^2^.

The total heat release reflects the amount of heat released during the entire combustion process of a material under specific thermal radiation conditions. Figure 10 presents the total heat release of MSA, DSA, and TSA at 15 kW/m^2^, 20 kW/m^2^, 25 kW/m^2^, and 35 kW/m^2^, respectively. The total heat release initially increases over time and remains constant until the end of the combustion. It is evident that at a specific heat flux, the THR of the three SAs follows a particular order, i.e., TSA > DSA > MSA. At a heat flux of 35 kW/m^2^, the largest total heat release of TSA, DSA, and MSA is 9.01 MJ/m^2^, 7.72 MJ/m^2^, and 6.57 MJ/m^2^, respectively. In terms of total heat release, TSA still has the greatest thermal hazard among the three methylsilyl-modified SAs.

**Figure 11:**
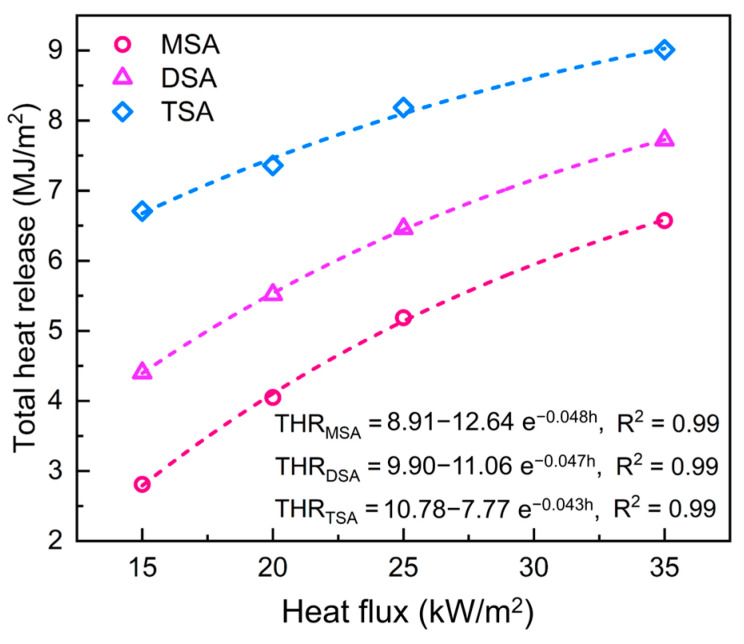
The variations in total heat release versus the heat flux. The fitted equations are presented above, in which THR is the total heat release and h is the heat flux.

Figure 11 shows the variation between total heat release and heat flux. It confirms that the total heat release of all three methylsilyl-modified SAs increases as per a negative exponent with the heat flux. According to the fitting functions, the maximal total heat release of MSA, DSA, and TSA is 8.91 MJ/m^2^, 9.90 MJ/m^2^ and 10.78 MJ/m^2^, respectively. Their minimums are calculated along with the critical heat flux and are 2.68 MJ/m^2^, 4.15 MJ/m^2^, and 6.45 MJ/m^2^. A comparison of the maximums and minimums of total heat release further indicates that TSA releases the most heat and has the most severe thermal hazard while MSA releases a moderate amount of heat and is relatively thermally safe.

#### 2.3.2. Fire Performance Index

**Figure 12:**
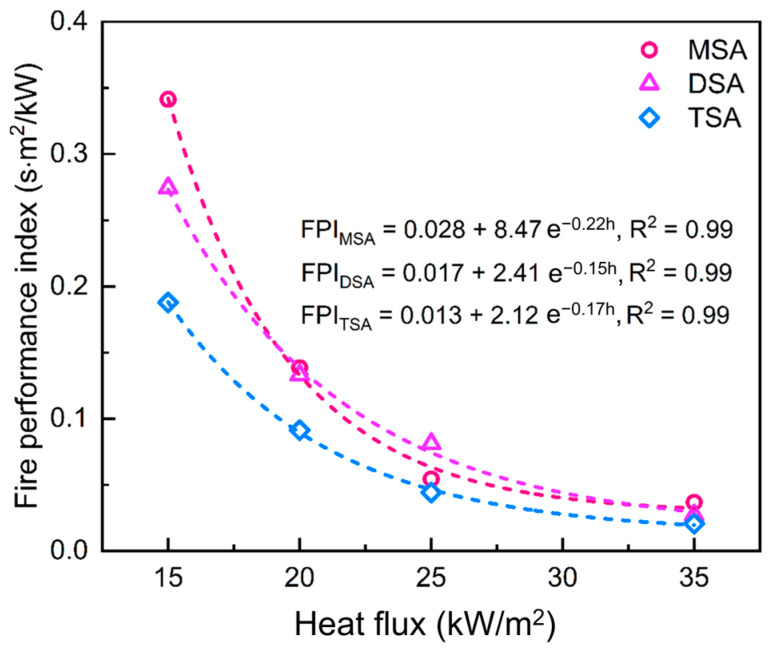
Variations in fire performance index versus heat flux. The fitted equations are presented above, in which FPI is the fire performance index and h is the heat flux.

Another important combustion parameter is calculated to further evaluate the fire hazard of methylsilyl-modified SAs, i.e., the fire performance index (FPI). The FPI reflects the propensity for flashover in a full-scale fire, defined as the ratio of TTI to pHRR [8]. A smaller FPI means a greater propensity for flashover [49]. Figure 12 presents the change in the FPI with the heat flux of the three methylsilyl-modified SAs. We found that the FPIs all exponentially decreased with an increase in heat flux. As the heat flux increased to 35 kW/m^2^, the FPI of MSA, DSA, and TSA decreased to 0.037 s·m^2^/kW, 0.027 s·m^2^/kW, and 0.021 s·m^2^/kW, respectively. With a further increase in heat flux, the minimum FPI reached as low as 0.028 s·m^2^/kW, 0.017 s·m^2^/kW, and 0.013 s·m^2^/kW. All these obtained FPIs are lower than that of commercial expanded polystyrene (EPS, with an FPI of ~0.035 s·m^2^/kW) [50], which indisputably indicates a non-negligible fire hazard in the methylsilyl-modified SAs. Furthermore, the FPI in TSA is evidently lower than that in DSA and MSA at a specific heat flux, suggesting that TSA has a relatively larger fire risk than the other two SAs.

### 2.4. Combustion Process and Mechanism

**Figure 13:**
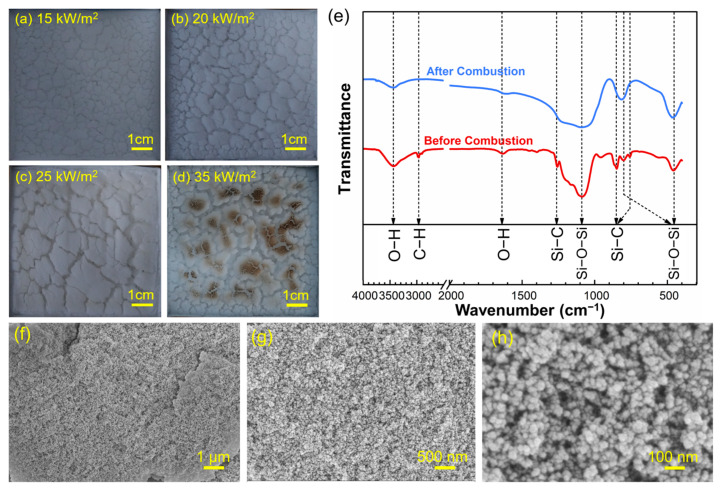
(**a**–**d**) Photographs of the residual char from TSA after the cone test at different heat fluxes: (**e**) the FTIR before and after the test, and (**f**–**h**) the microstructure of residual char from TSA.

Taking TSA as an example, significant changes were observed in the morphology, FTIR spectrum, and residue structure before and after the cone calorimeter test (Figure 13). Macroscopically, the surface showed sintering marks and exhibited a scorched yellow color at 35 kW/m^2^. Cracks widened and aggregated block areas increased with the radiation power. On the one hand, the greater the thermal radiation power, the more combustible gases can be released outward at the same time. On the other hand, the mutual condensation and cross-linking between the silanol groups formed by pyrolysis cause the aerogel particles to further condense together [51]. In the FTIR spectra of Figure 13e, before the cone test, characteristic peaks of the modified groups were present, including Si-C at 1257 cm^−1^, 847 cm^−1^, and 757 cm^−1^, and C-H between 2980–2900 cm^−1^. After the test, these peaks disappeared. As shown in Figure 13f, at a scale of 1 μm, distinct cracks are visible, and it is hypothesized that these cracks might be attributed to the sintering process during combustion. Upon further magnification, the skeletal structure was found to have collapsed, resulting in the formation of continuous and dense spherical particles. These changes provide insights into the combustion process and mechanism.

**Figure 14:**
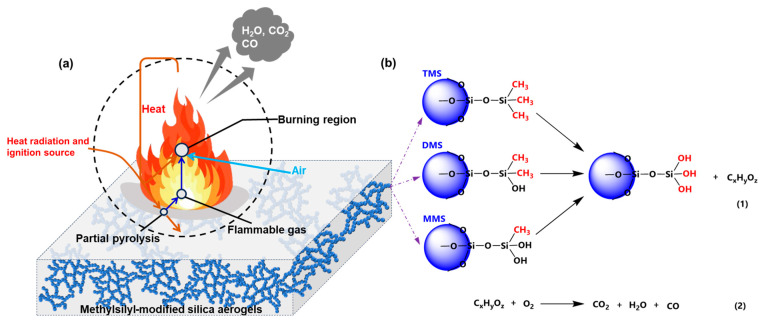
(**a**) Combustion process and (**b**) mechanism of methylsilyl-modified SAs.

The combustion process and mechanism of the three methylsilyl-modified SAs are further summarized in Figure 14. Based on the previous discussion on surface chemistry and combustion characteristics, the methylsilyl groups introduced for hydrophobization are considered the primary factor influencing the combustion behavior of these aerogels.

Under specific external heat radiation, the methylsilyl-modified SAs begin to locally decompose, releasing heat (evident from the exothermic peaks in the DSC curves) and flammable volatile components (denoted as CxHyOz) once the temperatures reach the thermal decomposition onset (approximately 245 °C–275 °C [43]). As the concentration of these volatiles increases, ignition occurs when an external source such as an electric spark is introduced. The flames spread rapidly across the surface of the aerogel powders and intensify. The combustion products are primarily water, carbon dioxide, and carbon monoxide. As the combustion progresses, the release of flammable volatiles gradually diminishes, leading to the weakening and eventual extinction of the flames. The simplified chemical reaction equations of combustion are shown in Figure 14b.

From a combustion process perspective, no significant differences are observed among the three methylsilyl-modified SAs. The primary variation in thermal hazard arises from the heat release behavior, which is influenced by both the onset and peak temperatures of thermal decomposition. These factors are tied to the specific type of methylsilyl groups present on the aerogel skeletons [43]. Additionally, the quantity of methylsilyl groups also affects the heat release, as demonstrated by TG and gross calorific value tests. Thus, the differences in combustion behavior among the methylsilyl-modified SAs are primarily determined by the type and quantity of methylsilyl groups.

## 3. Conclusions

This study comprehensively investigated the combustion characteristics and thermal hazards of three types of methylsilyl-modified silica aerogels (MSA, DSA, and TSA). The key findings of this research are summarized as follows:

Combustion behavior: The combustion process of all three methylsilyl-modified aerogels followed a similar pattern, characterized by smoldering, violent burning, and burning-down stages. However, TSA exhibited the highest heat release rate, indicating a greater fire hazard compared to DSA and MSA. This behavior can be attributed to the higher quantity of methyl groups grafted onto the silica skeleton.

Thermal stability and hazard: The thermal decomposition onset temperature for these aerogels ranged from 245 °C to 275 °C, with variations depending on the type of methylsilyl group. TSA, having the largest number of methyl groups, also showed the highest heat release and fastest fire spread rate. The thermal hazards of these aerogels were found to be directly correlated with the type and quantity of methylsilyl groups introduced during modification.

Fire performance: The fire performance index values indicated that TSA poses the most significant fire risk among the tested aerogels, with the lowest FPI value, followed by DSA and MSA. This highlights the importance of considering the quantity and type of methylsilyl groups in mitigating fire risk for practical applications.

In conclusion, while methylsilyl modification enhances the hydrophobicity of silica aerogels, it also increases their flammability and thermal hazard. Therefore, the trade-off between improved hydrophobic properties and heightened fire risk must be carefully considered in thermal insulation applications. Future studies could explore potential flame-retardant modifications to mitigate the fire hazards posed by methylsilyl-modified aerogels.

## 4. Materials and Methods

### 4.1. Raw Materials

Tetraethoxysilane (TEOS, Si(OC_2_H_5_)_4_, m(SiO_2_) ≥ 28.4%) was used as a precursor, and trimethylchlorosilane (TMCS, C_3_H_9_ClSi, 98%), dimethyldichlorosilane (DMDCS, C_2_H_6_Cl_2_Si, 98%), and methyltrichlorosilane (MTCS, CH_3_Cl_3_Si, 99%) were used as the surface modifiers, respectively. All reagents were bought from Aladdin (Shanghai, China). Ethanol (EtOH, C_2_H_5_OH 99.7%), hydrochloric acid (HCl, 36.5%), ammonium hydroxide (NH_3_·H_2_O, 26.5–28.5%), and n-hexane (C_6_H_14_, 97%) were used as the solvent, acid and base catalysts, respectively, which were from Sinopharm Chemical Reagent Co., Ltd., Shanghai, China.

### 4.2. Aerogel Preparation

**Figure 15:**
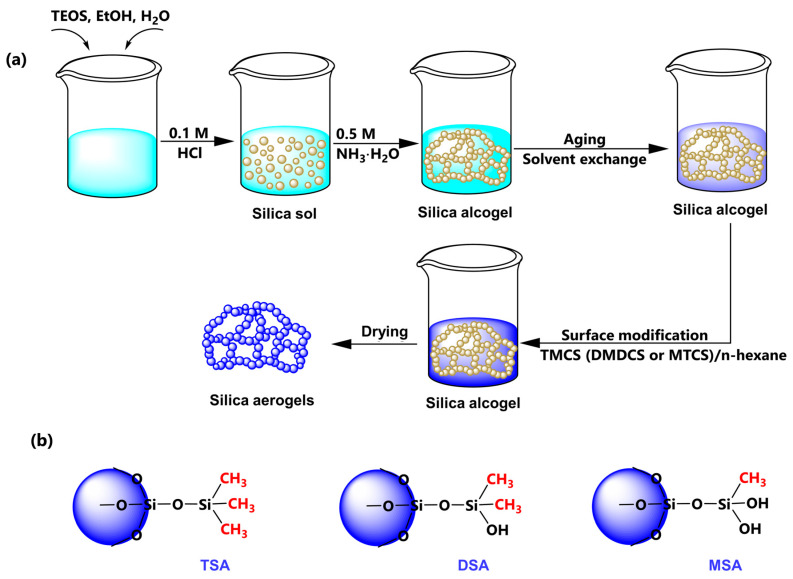
(**a**) Preparation flow chart and (**b**) difference of various methylsilyl-modified SAs.

The detailed preparation process of hydrophobic SiO_2_ aerogels is given in Figure 15. TEOS, EtOH, deionized water, and 0.1 mol/L of hydrochloric acid (TEOS:EtOH:H_2_O:HCl = 1:9.6:2.16:1.6 × 10^−3^) were mixed in a beaker, stirred for 5 min, and then placed in a water bath at 45 °C for 10 h to hydrolyze completely. Subsequently, 0.5 mol/L NH_3_·H_2_O was added to the beaker and stirred for 3 min, and the beaker was placed in a 45 °C water bath for 4 h until gelation. After gelation, the gels were aged and exchanged with an appropriate amount of EtOH or n-hexane for 18 h, respectively. The 10% TMCS (or DMDCS, MTCS)/n-hexane was employed to the modified gels at 45 °C for 12 h. Finally, the methylsilyl-modified SAs were obtained after drying at 120 °C for 8 h. Note that the SAs modified with TMCS, DMDCS, and MTCS were renamed TSA, DSA, and MSA, respectively.

### 4.3. Basic Characterization

The bulk density (*ρ_b_*) was evaluated by the ratio of mass to volume, and the porosity was calculated as follows:

(1) $$Porosity=\left(1-\frac{\rho_{b}}{\rho_{s}}\right)\times 100\%$$

where *ρ_s_* is the skeletal density of SAs, about 2.2 g/cm^3^. The prepared SAs were ground into 200 mesh powder, and the tap density was measured by a tap density meter at a rotation speed of 300 r/min and a vibration time of 10 min.

The microstructures of SAs were observed with a scanning electron microscope (SEM, Sigma 300, Zeiss, Oberkochen, Germany). The pore structure was characterized using the nitrogen sorption measurement (AUTOSORB IQ, Quantachrome, FL, USA), the Brunauer–Emmett–Teller (BET) method was used for the apparent surface area, and the Barrett–Joyner–Halenda (BJH) method was used for the pore size distribution. To obtain more comprehensive pore structure parameters, the pore volume (*V_pore_*) and pore diameter (*D_pore_*) were also calculated by using Equations (2) and (3):

(2) $$V_{\mathrm{pore}}=\frac{1}{\rho_{b}}-\frac{1}{\rho_{s}}$$

(3) $$D_{\mathrm{pore}}=\frac{4V_{\mathrm{pore}}}{S_{BET}}$$

where *ρ_b_* is the bulk density, *ρ_s_* is the density of the aerogel skeleton, about 2.2 g/cm^3^, and *S_BET_* is the apparent surface area obtained by the BET method.

Fourier transform infrared spectroscopy (FTIR, Nicolet iS50, Thermo Fisher Scientific, Waltham, MA, USA) was used to characterize the chemical bonds or functional groups. The contact angles were measured with a contact angle meter (ASR-7055, AISRY, Dongguan, China), which are then used to characterize the hydrophobicity of the methylsilyl-modified SAs. The thermal insulation property was measured using a thermal conductivity tester (TC3000E, XIATECH, Xi’an, China).

### 4.4. Combustion Tests

Combustion characteristics were studied by using a cone calorimeter (CCT, MOTIS, Kunshan, China) as per the ISO 5660-1:2015 standard [52]. The methylsilyl-modified SA powders of 200 mesh were uniformly tiled in a specimen holder of 80 mm × 80 mm × 10 mm and the heat fluxes were set at 15 kW/m^2^, 20 kW/m^2^, 25 kW/m^2^, and 35 kW/m^2^, respectively, in the tests. Each sample was tested three times with a typical repeatability of 10% [50]. In addition, a digital camera (SONY FDR-AX700, Tokyo, Japan) was used to record the combustion process and flame shapes. The video resolution was 1920 × 1080 ppi and the frame rate was 25 frames/s. The fire spread speed was obtained through post-processing the obtained videos. The combustion parameters, including heat release rate (HRR), peak of heat release rate (PHRR), time to ignition (TTI), time to PHRR (TTPHRR) and total heat release (THR), were obtained directly from the cone calorimeter.

TG-DSC analyses were performed to identify the thermal stability of SAs by using a simultaneous thermogravimetry analyzer (SDT Q600, Waters, Milford, CT, USA) with a heating rate of 10 °C/min from room temperature to 1000 °C in air. Gross calorific value (GCV) was determined by an oxygen bomb calorimeter (AM-C1009, Yuanfa, Changsha, China).

## Figures and Tables

**Table 1:** Pore size parameter of MSA, DSA, and TSA.

Sample	BET Surface Area (m^2^/g)	Pore Volume (cm^3^/g)	Average Pore Size (nm)	V_pore_ (cm^3^/g)	D_pore_ (nm)
MSA	773	3.0	3.8	6.6	34.2
DSA	878	3.9	3.1	8.5	38.8
TSA	1002	2.6	3.0	10.5	41.9

## Data Availability

The original contributions presented in the study are included in the article, further inquiries can be directed to the corresponding authors.

## References

[1] Zhang X., Ni X., Li C., You B., Sun G. (2020). Co-Gel Strategy for Preparing Hierarchically Porous Silica/Polyimide Nanocomposite Aerogel with Thermal Insulation and Flame Retardancy. J. Mater. Chem. A.

[2] Liu Z., Lyu J., Fang D., Zhang X. (2019). Nanofibrous Kevlar Aerogel Threads for Thermal Insulation in Harsh Environments. ACS Nano.

[3] Soleimani Dorcheh A., Abbasi M.H. (2008). Silica Aerogel; Synthesis, Properties and Characterization. J. Mater. Process. Technol..

[4] Kistler S.S. (1931). Coherent Expanded Aerogels and Jellies. Nature.

[5] Maleki H. (2016). Recent Advances in Aerogels for Environmental Remediation Applications: A Review. Chem. Eng. J..

[6] Maleki H., Durães L., Portugal A. (2014). An Overview on Silica Aerogels Synthesis and Different Mechanical Reinforcing Strategies. J. Non-Cryst. Solids.

[7] Ma Q., Liu Y., Dong Z., Wang J., Hou X. (2015). Hydrophobic and Nanoporous Chitosan-Silica Composite Aerogels for Oil Absorption. J. Appl. Polym. Sci..

[8] Yin L., Xu J., Zhang B., Wang L., Tao W., Teng X., Ning W., Zhang Z. (2022). A Facile Fabrication of Highly Dispersed CeO_2_/SiO_2_ Aerogel Composites with High Adsorption Desulfurization Performance. Chem. Eng. J..

[9] Bruzzoniti M.C., Appendini M., Rivoira L., Onida B., Bubba M.D., Jana P., Sorarù G.D. (2018). Polymer-derived ceramic aerogels as sorbent materials for the removal of organic dyes from aqueous solutions. J. Am. Ceram. Soc..

[10] Villasmil W., Fischer L.J., Worlitschek J. (2019). A Review and Evaluation of Thermal Insulation Materials and Methods for Thermal Energy Storage Systems. Renew. Sustain. Energy Rev..

[11] Tang G.H., Zhao Y., Guo J.F. (2016). Multi-Layer Graded Doping in Silica Aerogel Insulation with Temperature Gradient. Int. J. Heat Mass Transf..

[12] Çok S.S., Gizli N. (2020). Hydrophobic Silica Aerogels Synthesized in Ambient Conditions by Preserving the Pore Structure via Two-Step Silylation. Ceram. Int..

[13] Zhao Y., Liang Y., Zhao X., Jia Q., Li H. (2011). Preparation and Microstructure of CuO-CoO-MnO/SiO_2_ Nanocomposite Aerogels and Xerogels as Catalyst Carriers. Prog. Nat. Sci. Mater. Int..

[14] Zhang Q., Chen C., Wang M., Cai J., Xu J., Xia C. (2011). Facile Preparation of Highly-Dispersed Cobalt-Silicon Mixed Oxide Nanosphere and Its Catalytic Application in Cyclohexane Selective Oxidation. Nanoscale Res. Lett..

[15] Ulker Z., Erkey C. (2014). An Emerging Platform for Drug Delivery: Aerogel Based Systems. J. Control. Release.

[16] García-González C.A., Sosnik A., Kalmár J., De Marco I., Erkey C., Concheiro A., Alvarez-Lorenzo C. (2021). Aerogels in Drug Delivery: From Design to Application. J. Control. Release.

[17] Almeida C.M.R., Ghica M.E., Ramalho A.L., Durães L. (2021). Silica-Based Aerogel Composites Reinforced with Different Aramid Fibres for Thermal Insulation in Space Environments. J. Mater. Sci..

[18] Randall J.P., Meador M.A.B., Jana S.C. (2011). Tailoring Mechanical Properties of Aerogels for Aerospace Applications. ACS Appl. Mater. Interfaces.

[19] Bergmann Becker P.F., Effting C., Schackow A. (2022). Lightweight Thermal Insulating Coating Mortars with Aerogel, EPS, and Vermiculite for Energy Conservation in Buildings. Cem. Concr. Compos..

[20] He F., Qi Z., Zhen W., Wu J., Huang Y., Xiong X., Zhang R. (2019). Thermal Conductivity of Silica Aerogel Thermal Insulation Coatings. Int. J. Thermophys..

[21] Kovács Z., Csík A., Lakatos Á. (2023). Thermal Stability Investigations of Different Aerogel Insulation Materials at Elevated Temperature. Therm. Sci. Eng. Prog..

[22] Li C., Chen Z., Dong W., Lin L., Zhu X., Liu Q., Zhang Y., Zhai N., Zhou Z., Wang Y. (2021). A Review of Silicon-Based Aerogel Thermal Insulation Materials: Performance Optimization through Composition and Microstructure. J. Non-Cryst. Solids.

[23] Wu Q., Chen Z., Ding Y., Yin L., Yang M., Erişen D.E., Liu T., Li M., Yang L., Cui S. (2024). A Flexible Double Network Aerogel Reinforced by SiO_2_/ZrO_2_ Fibers Paper with Excellent Thermal Insulation at High-Temperature. Ceram. Int..

[24] Zhan W., Feng X., Zhang Q., Chen L., Li L., Kong Q., Shi F., Chen M., Du D., Jiang J. (2024). Effects of Silica Aerogel Particles on Performance of the Coatings for New Energy Vehicle Battery Packs. J. Dispers. Sci. Technol..

[25] Huang S., Wu X., Li Z., Shi L., Zhang Y., Liu Q. (2020). Rapid Synthesis and Characterization of Monolithic Ambient Pressure Dried MTMS Aerogels in Pure Water. J. Porous. Mater..

[26] Zhao S., Siqueira G., Drdova S., Norris D., Ubert C., Bonnin A., Galmarini S., Ganobjak M., Pan Z., Brunner S. (2020). Additive Manufacturing of Silica Aerogels. Nature.

[27] Gheonea R., Crasmareanu E.C., Plesu N., Sauca S., Simulescu V., Ilia G. (2017). New Hybrid Materials Synthesized with Different Dyes by Sol-Gel Method. Adv. Mater. Sci. Eng..

[28] Macarie L., Pekar M., Simulescu V., Plesu N., Iliescu S., Ilia G., Tara-Lunga-Mihali M. (2017). Properties in Aqueous Solution of Homo- and Copolymers of Vinylphosphonic Acid Derivatives Obtained by UV-Curing. Macromol. Res..

[29] Wu X., Zhong K., Ding J., Shen X., Cui S., Zhong Y., Ma J., Chen X. (2020). Facile Synthesis of Flexible and Hydrophobic Polymethylsilsesquioxane Based Silica Aerogel via the Co-Precursor Method and Ambient Pressure Drying Technique. J. Non-Cryst. Solids.

[30] Yao C., Dong X., Gao G., Sha F., Xu D. (2021). Microstructure and Adsorption Properties of MTMS/TEOS Co-Precursor Silica Aerogels Dried at Ambient Pressure. J. Non-Cryst. Solids.

[31] Sorour M.H., Hani H.A., Al-Bazedi G.A., EL-Rafei A.M. (2016). Hydrophobic Silica Aerogels for Oil Spills Clean-up, Synthesis, Characterization and Preliminary Performance Evaluation. J. Porous. Mater..

[32] Sert Çok S., Koç F., Gizli N. (2021). Lightweight and Highly Hydrophobic Silica Aerogels Dried in Ambient Pressure for an Efficient Oil/Organic Solvent Adsorption. J. Hazard. Mater..

[33] Gurav J.L., Nadargi D.Y., Rao A.V. (2008). Effect of Mixed Catalysts System on TEOS-Based Silica Aerogels Dried at Ambient Pressure. Appl. Surf. Sci..

[34] Panyo C., Wannagon A., Chimupala Y., Pearce J.T.H., Nuntiya A. (2023). Silica Aerogel from Sugarcane Bagasse Ash Incorporated Cementitious Thermal Insulation Composites. Mater. Lett..

[35] Rao A.V., Kalesh R.R. (2004). Organic Surface Modification of TEOS Based Silica Aerogels Synthesized by Co-Precursor and Derivatization Methods. J. Sol-Gel Sci. Technol..

[36] Rao A.V., Kulkarni M.M., Pajonk G.M., Amalnerkar D.P., Seth T. (2003). Synthesis and Characterization of Hydrophobic Silica Aerogels Using Trimethylethoxysilane as a Co-Precursor. J. Sol-Gel Sci. Technol..

[37] Luo Y., Li Z., Zhang W., Yan H., Wang Y., Li M., Liu Q. (2019). Rapid Synthesis and Characterization of Ambient Pressure Dried Monolithic Silica Aerogels in Ethanol/Water Co-Solvent System. J. Non-Cryst. Solids.

[38] Li Z., Huang S., Shi L., Li Z., Liu Q., Li M. (2019). Reducing the Flammability of Hydrophobic Silica Aerogels by Doping with Hydroxides. J. Hazard. Mater..

[39] Ghazi Wakili K., Remhof A. (2017). Reaction of Aerogel Containing Ceramic Fibre Insulation to Fire Exposure. Fire Mater..

[40] He S., Huang Y., Chen G., Feng M., Dai H., Yuan B., Chen X. (2019). Effect of Heat Treatment on Hydrophobic Silica Aerogel. J. Hazard. Mater..

[41] Zhang W., Li Z., Shi L., Li Z., Luo Y., Liu Q., Huang R. (2019). Methyltrichlorosilane Modified Hydrophobic Silica Aerogels and Their Kinetic and Thermodynamic Behaviors. J. Sol-Gel Sci. Technol..

[42] Li Z., Zhang Y., Huang S., Wu X., Shi L., Liu Q. (2020). Thermal Stability and Pyrolysis Characteristics of MTMS Aerogels Prepared in Pure Water. J. Nanopart. Res..

[43] Li Z., Wang Y., Wu X., Liu Q., Li M., Shi L., Cheng X. (2023). Surface Chemistry, Skeleton Structure and Thermal Safety of Methylsilyl Modified Silica Aerogels by Heat Treatment in an Argon Atmosphere. J. Non-Cryst. Solids.

[44] Han M.L., Wei X.L., Zhang J.C., Liu Y., Tang X., Li P., Liu Z.Y. (2022). Influence of Structural Damage on Evaluation of Microscopic Pore Structure in Marine Continental Transitional Shale of the Southern North China Basin: A Method Based on the Low-Temperature N_2_ Adsorption Experiment. Pet. Sci..

[45] Estella J., Echeverría J.C., Laguna M., Garrido J.J. (2007). Effects of Aging and Drying Conditions on the Structural and Textural Properties of Silica Gels. Microporous Mesoporous Mater..

[46] Wang X., Liu Q., Li M., Chen Z., Cheng X., Wu X., Li Z. (2024). Insights into the Thermal Safety of Ambient Pressure Dried Hydrophobic Montmorillonite/Silica Aerogel Composites. Ceram. Int..

[47] Chen H.-B., Wang Y.-Z., Sánchez-Soto M., Schiraldi D.A. (2012). Low Flammability, Foam-like Materials Based on Ammonium Alginate and Sodium Montmorillonite Clay. Polymer.

[48] Zanganeh J., Moghtaderi B., Ishida H. (2013). Combustion and Flame Spread on Fuel-Soaked Porous Solids. Prog. Energy Combust. Sci..

[49] Li Z., Cheng X., Gong L., Liu Q., Li S. (2018). Enhanced Flame Retardancy of Hydrophobic Silica Aerogels by Using Sodium Silicate as Precursor and Phosphoric Acid as Catalyst. J. Non-Cryst. Solids.

[50] Chen H.-B., Wang Y.-Z., Schiraldi D.A. (2014). Preparation and Flammability of Poly(Vinyl Alcohol) Composite Aerogels. ACS Appl. Mater. Interfaces.

[51] Bippus L., Jaber M., Lebeau B., Schleich D., Scudeller Y. (2014). Thermal Conductivity of Heat Treated Mesoporous Silica Particles. Microporous Mesoporous Mater..

[52] (2015). Heat Release Rate (Cone Calorimeter Method) and Smoke Production Rate (Dynamic Measurement).

